# Supplementary material for: Household hardships and responses to COVID-19 pandemic-related shocks in Eastern Ethiopia
Source: BMC Public Health. 2023 Oct 25;23:2086. doi: 10.1186/s12889-023-16982-0 (PMC10598954; doi:10.1186/s12889-023-16982-0)
Supplement: Supplementary file 1 — Additional file 1. [file 12889_2023_16982_MOESM1_ESM.zip › Appendix A/Appendix A.pdf]

Table A.1: Principal Components Factor Analysis of Hardship Variables

**Variable**	**Factor Patterns**
Job Loss	0.60
Business Closure	0.87
Farm Disruption	0.79
Livestock Disruption	0.92
Fishing Disruption	0.80
Output Disruption	0.87
Eigenvalue	4.00
Explained Variance	40%
