# Supplementary material for: Household hardships and responses to COVID-19 pandemic-related shocks in Eastern Ethiopia
Source: BMC Public Health. 2023 Oct 25;23:2086. doi: 10.1186/s12889-023-16982-0 (PMC10598954; doi:10.1186/s12889-023-16982-0)
Supplement: Supplementary file 1 — Additional file 1. [file 12889_2023_16982_MOESM1_ESM.zip › Appendix B/Appendix B.docx]

**Appendix B**

A wealth index was generated based on a collection of assets and construction materials for the main dwelling of a given household. To generate the index, we followed recommendations from the DHS and the World Food Programme (WFP) that summarize steps for calculating an asset-based wealth index [48-50], including coding instructions for Stata [51], and an adaptation of this coding process implemented in R [30, 52]. Using these documents to guide us, we generated our wealth index by identifying a list of household assets for inclusion in our index computation. We recoded all household assets into dichotomous variables and recoded dwelling materials into improved vs. non-improved dichotomous variables based on DHS recommendations. We divided our sample into rural and urban sub-samples and assessed level of representation of a given variable within the rural and urban sub-samples (per WFP recommendations, a given variable was included in further calculations if percent ownership ranged between 5 and 95 percent). We then employed principal components analysis with varimax rotation to calculate component scores for those households living in either rural or urban areas, which explained 45 percent of the variation in both sub-samples, and extracted and combined the PCA scores of the first component from the urban and rural sub-samples; and finally organized the scores into wealth quintiles to generate a composite asset index. The distribution of assets owned by households as well as the materials used for constructing a household’s residence are presented in Figure B.1.

Figure B.1. Distribution of Households' Asset Ownership and Dwelling Construction Materials. These assets and construction materials were used as inputs for calculating the wealth index.
