# Supplementary figures and images for: Household hardships and responses to COVID-19 pandemic-related shocks in Eastern Ethiopia
Source: BMC Public Health. 2023 Oct 25;23:2086. doi: 10.1186/s12889-023-16982-0 (PMC10598954; doi:10.1186/s12889-023-16982-0)

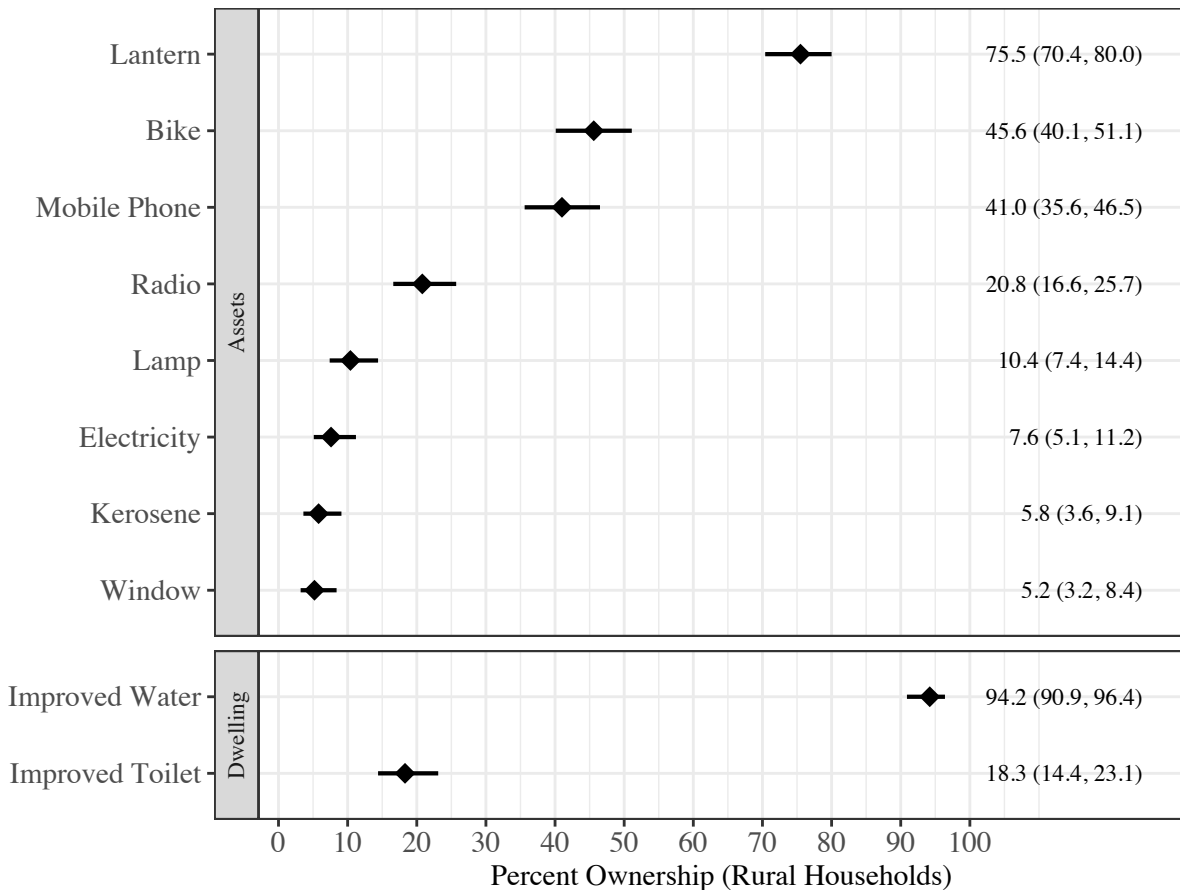

Supplement: Supplementary file 1 — Additional file 1. [file 12889_2023_16982_MOESM1_ESM.zip › Appendix B/Wealth_Index_Assets_Distribution_By_Category_Rural.pdf]

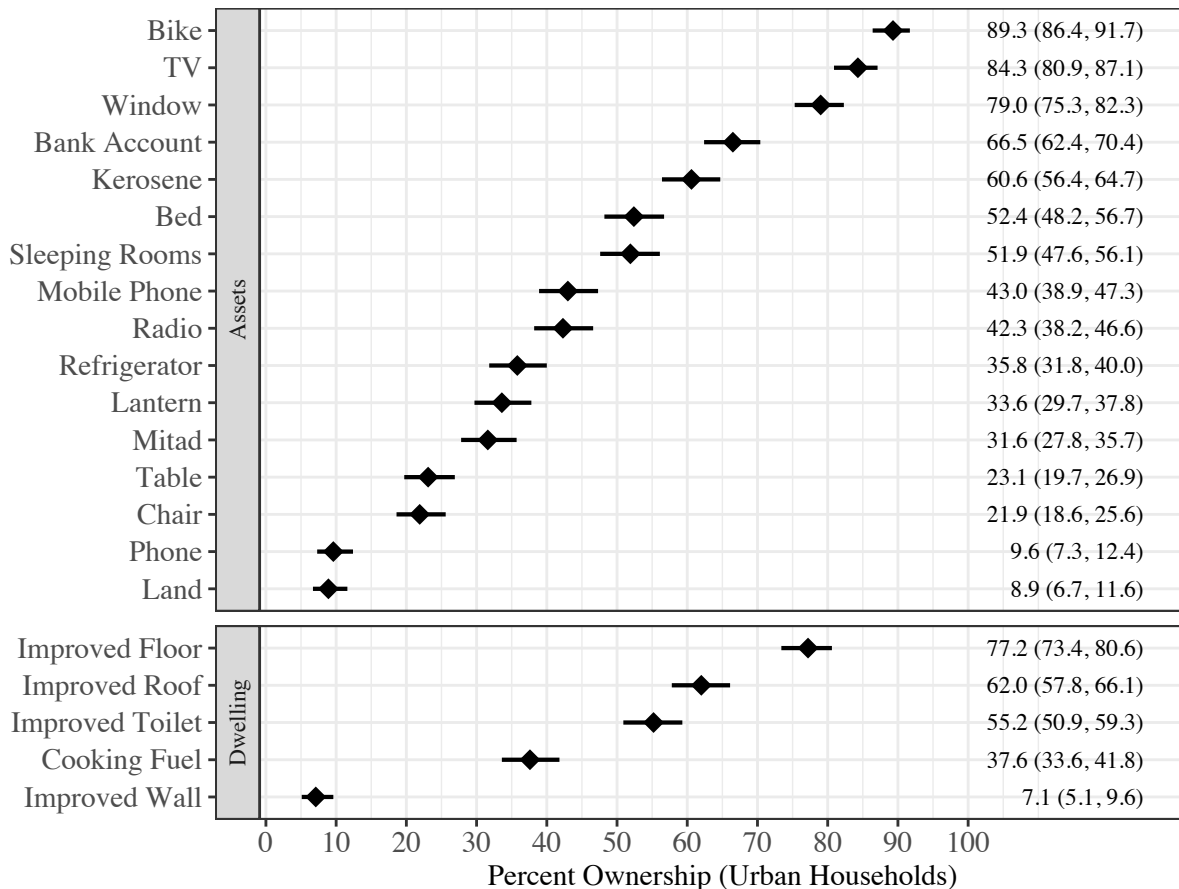

Supplement: Supplementary file 1 — Additional file 1. [file 12889_2023_16982_MOESM1_ESM.zip › Appendix B/Wealth_Index_Assets_Distribution_By_Category_Urban.pdf]

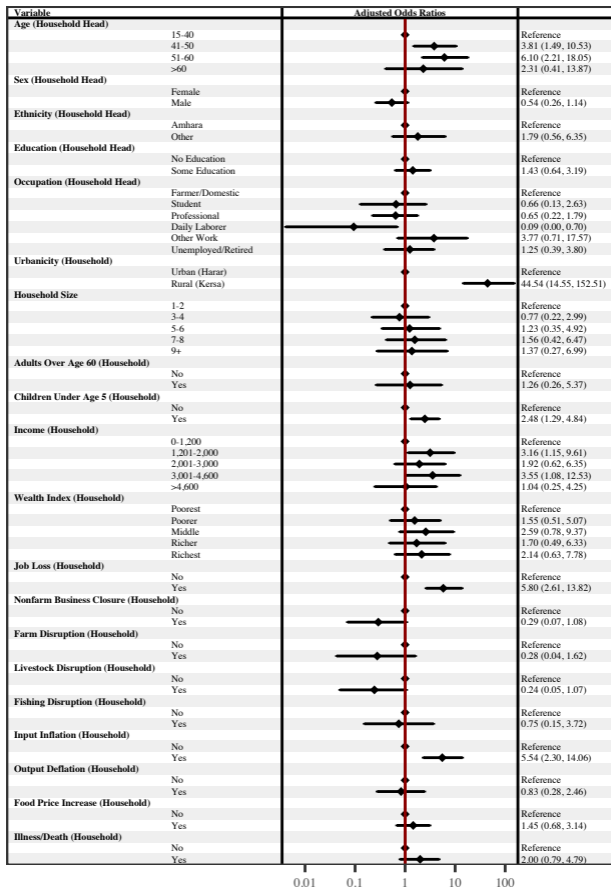

Supplement: Supplementary file 1 — Additional file 1. [file 12889_2023_16982_MOESM1_ESM.zip › Appendix C/Adjusted_Plot_FF_Borrow.pdf]

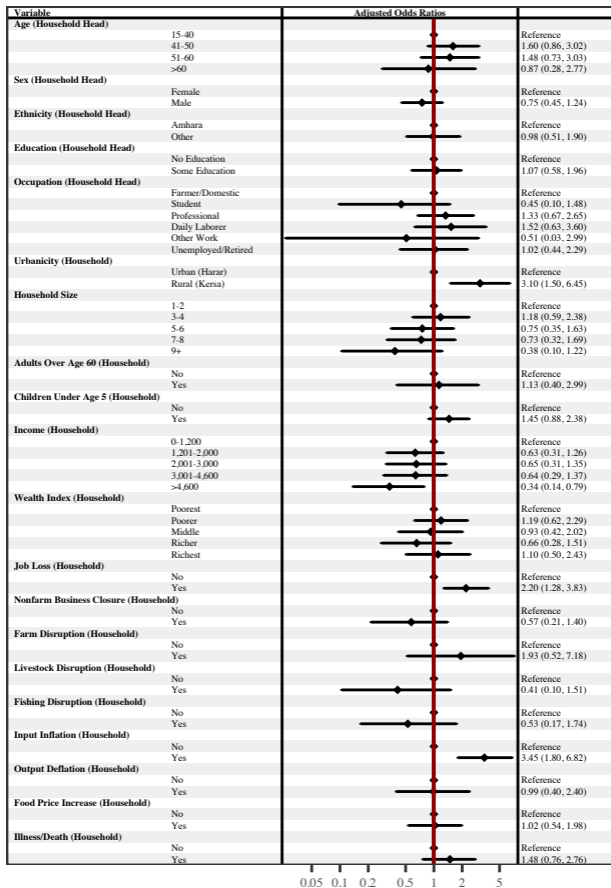

Supplement: Supplementary file 1 — Additional file 1. [file 12889_2023_16982_MOESM1_ESM.zip › Appendix C/Adjusted_Plot_FF_Help.pdf]

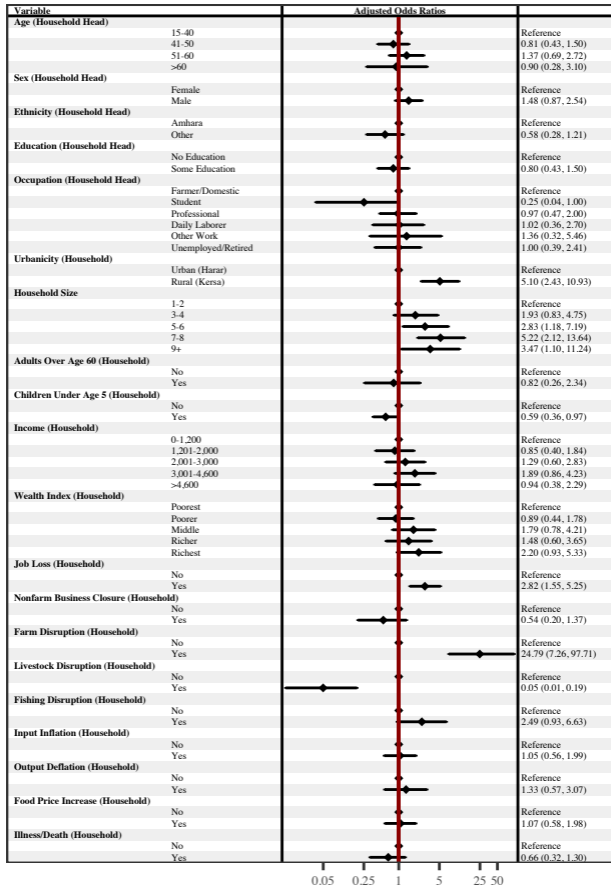

Supplement: Supplementary file 1 — Additional file 1. [file 12889_2023_16982_MOESM1_ESM.zip › Appendix C/Adjusted_Plot_Generate_Income.pdf]

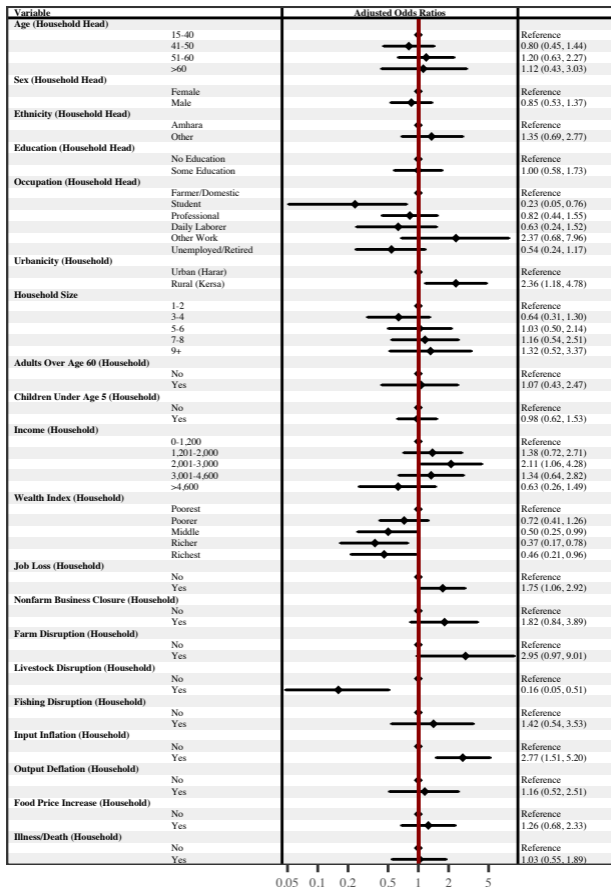

Supplement: Supplementary file 1 — Additional file 1. [file 12889_2023_16982_MOESM1_ESM.zip › Appendix C/Adjusted_Plot_Reduced_Food_Consumption.pdf]

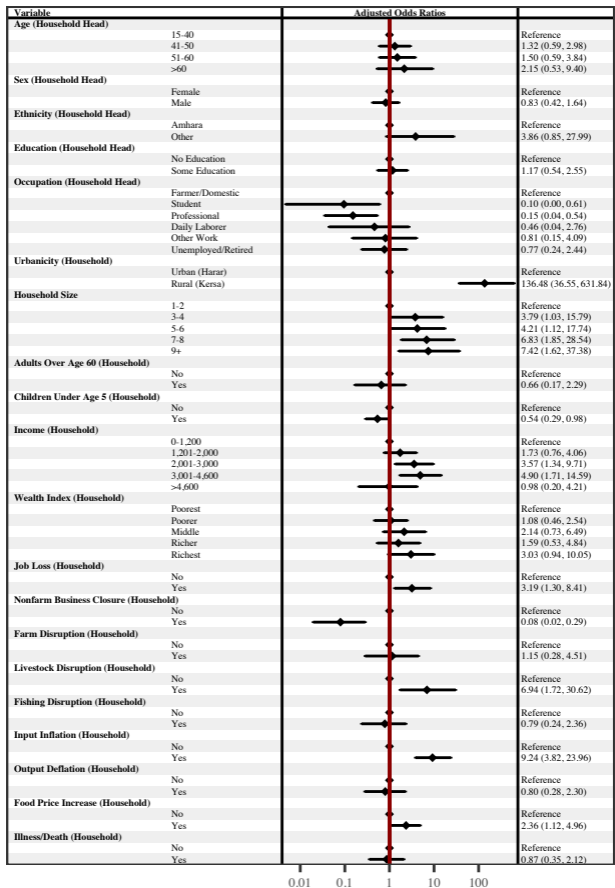

Supplement: Supplementary file 1 — Additional file 1. [file 12889_2023_16982_MOESM1_ESM.zip › Appendix C/Adjusted_Plot_Sell_Assets.pdf]
